# Comparison of commercial dosimetric software platforms in patients treated with ^177^Lu‐DOTATATE for peptide receptor radionuclide therapy

**DOI:** 10.1002/mp.14375

**Published:** 2020-07-31

**Authors:** Erick Mora‐Ramirez, Lore Santoro, Emmanuelle Cassol, Juan C. Ocampo‐Ramos, Naomi Clayton, Gunjan Kayal, Soufiane Chouaf, Dorian Trauchessec, Jean‐Pierre Pouget, Pierre‐Olivier Kotzki, Emmanuel Deshayes, Manuel Bardiès

**Affiliations:** ^1^ Centre de Recherches en Cancérologie de Toulouse UMR 1037 Toulouse F‐31037 France; ^2^ INSERM UMR 1037 Université Toulouse III Paul Sabatier Toulouse F‐31062 France; ^3^ Escuela de Física ‐ CICANUM Universidad de Costa Rica San José 11501‐2060 Costa Rica; ^4^ Département de Médecine Nucléaire Institut Régional du Cancer de Montpellier Montpellier F‐34298 France; ^5^ Département de Médecine Nucléaire Hôpitaux Toulouse Toulouse F‐31059 France; ^6^ Faculté de Médecine Rangueil Université Toulouse III Paul Sabatier Toulouse F‐31062 France; ^7^ SCK CEN Belgian Nuclear Research Centre Boeretang 200 Mol BE‐2400 Belgium; ^8^ Institut de Recherche en Cancérologie de Montpellier (IRCM) Inserm U1194 Université de Montpellier Institut Régional du Cancer de Montpellier (ICM) Montpellier F‐34298 France

**Keywords:** ^177^Lu‐DOTATATE, dosimetry, dosimetry software platforms, PRRT

## Abstract

**Purpose:**

The aim of this study was to quantitatively compare five commercial dosimetric software platforms based on the analysis of clinical datasets of patients who benefited from peptide receptor radionuclide therapy (PRRT) with ^177^Lu‐DOTATATE (LUTATHERA^®^).

**Methods:**

The dosimetric analysis was performed on two patients during two cycles of PRRT with ^177^Lu. Single photon emission computed tomography/computed tomography images were acquired at 4, 24, 72, and 192 h post injection. Reconstructed images were generated using Dosimetry Toolkit^®^ (DTK) from Xeleris™ and HybridRecon‐Oncology version_1.3_Dicom (HROD) from HERMES. Reconstructed images using DTK were analyzed using the same software to calculate time‐integrated activity coefficients (TIAC), and mean absorbed doses were estimated using OLINDA/EXM V1.0 with mass correction. Reconstructed images from HROD were uploaded into PLANET® OncoDose from DOSIsoft, STRATOS from Phillips, Hybrid Dosimetry Module™ from HERMES, and SurePlan™ MRT from MIM. Organ masses, TIACs, and mean absorbed doses were calculated from each application using their recommendations.

**Results:**

The majority of organ mass estimates varied by <9.5% between all platforms. The highest variability for TIAC results between platforms was seen for the kidneys (28.2%) for the two patients and the two treatment cycles. Relative standard deviations in mean absorbed doses were slightly higher compared with those observed for TIAC, but remained of the same order of magnitude between all platforms.

**Conclusions:**

When applying a similar processing approach, results obtained were of the same order of magnitude regardless of the platforms used. However, the comparison of the performances of currently available platforms is still difficult as they do not all address the same parts of the dosimetric analysis workflow. In addition, the way in which data are handled in each part of the chain from data acquisition to absorbed doses may be different, which complicates the comparison exercise. Therefore, the dissemination of commercial solutions for absorbed dose calculation calls for the development of tools and standards allowing for the comparison of the performances between dosimetric software platforms.

## 1 INTRODUCTION

Dosimetry contributes to the evaluation of the outcome and the optimization process of targeted radionuclide therapy. For example, peptide receptor radionuclide therapy (PRRT) optimization can be based on the evaluation of absorbed doses delivered to critical organs, such as kidneys and red or active bone marrow.^1^, ^2^, ^3^, ^4^, ^5^ Different approaches to clinical dosimetry have been proposed, based on whole body (WB) planar images,^6^, ^7^, ^8^, ^9^ single photon emission computed tomography/computed tomography (SPECT/CT) images,^10^, ^11^, ^12^, ^13^ and hybrid methods by combining WB planar images with one or two SPECT/CT scans.^14^, ^15^, ^16^, ^17^, ^18^

Imaging and data processing methodology have historically been predominantly specific to each institution, because there were no commercial software applications available to perform all aspects of clinical dosimetry. Therefore, academic/research institutions/hospitals developed in‐house dosimetric software, most of the time available only locally and not registered as medical devices (i.e., used in a research context only): DOSIMG,^19^ MABDOSE,^20^ DOSE3D,^21^ RMDP,^22^ VoxelDose,^23^ MrVoxel,^24^ OEDIPE,^25^ MINERVA,^26^ 3D‐RD,^27^ RAYDOSE,^28^ and NUKDOS,^29^ to name a few, belong to that category.

More recently, commercial software applications have been developed — some of which have or aim for FDA/EMA approval or CE marking, that is, are intended for use in a clinical environment. OLINDA/EXM (Version 1) is probably the most established and well‐known software that allows the computation of absorbed doses. GE Healthcare created the Dosimetry Toolkit^®^ (DTK) software^30^ as an option within its image/data processing workstation Xeleris^TM^. DTK recommends using OLINDA/EXM (Version 1) for the absorbed dose calculation step. HERMES developed the Hybrid Dosimetry Module™ (HDM) which integrates OLINDA/EXM version 2. The STRATOS software is part of the IMALYTICS by Phillips. PLANET^®^ Onco Dose (PDOSE) was introduced by DOSIsoft, and SurePlan™ MRT is proposed by MIM. Other software are available or have been announced recently (QDOSE^31^).

In fact, it is now possible for a nuclear medicine department to purchase such dosimetry software platforms. This will certainly encourage the development of routine clinical dosimetry, but calls for the appraisal of the characteristics of available commercial solutions.

According to the MIRD formalism,^32^ the mean absorbed dose D(r_T_, T_D_) to target tissue r_T_ over a defined dose‐integration period T_D_ (from 0 to infinity) after administration of the radioactive material can be expressed as:

$$D\left(r_{T},T_{D}\right)=\sum_{r_{s}}\tilde{A}\left(r_{s},T_{D}\right)S\left(r_{T}\leftarrow r_{s}\right)$$

where Ã(r_S_) is the time‐integrated activity (TIA) or total number of nuclear transformations in source tissue r_S_, and S(r_T_ ← r_S_) is the radionuclide‐specific quantity representing the mean absorbed dose in target tissue r_T_ per nuclear transformation in source tissue r_S_.

**Fig. 1:**
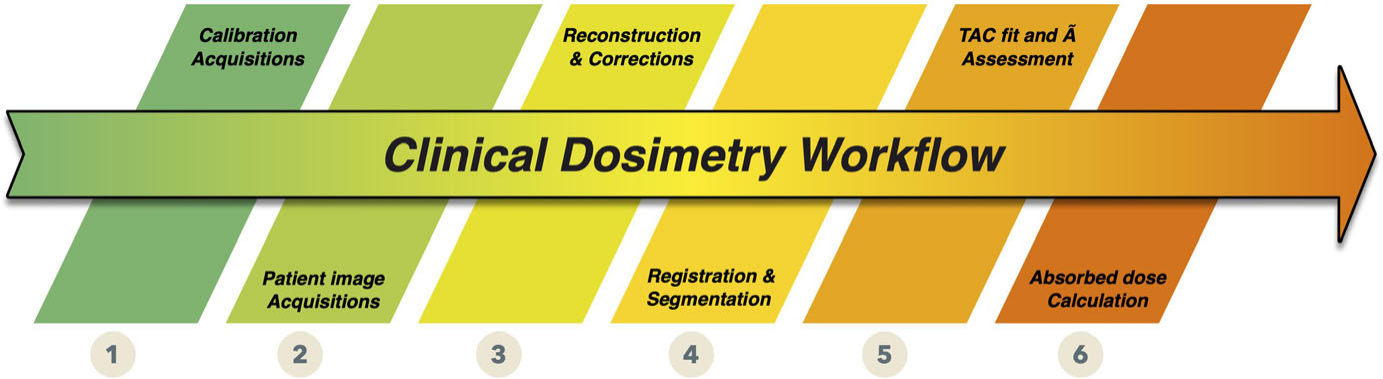
Clinical internal radionuclide dosimetry workflow. [Color figure can be viewed at wileyonlinelibrary.com]

This seemingly simple formulation hides in fact a series of operations, as illustrated in Fig. 1.

The first step in clinical dosimetry is that of the acquisition of calibration images. This is an individualized step because each dosimetry software platforms application requires calibration factors obtained according to specific acquisition protocols. Calibration (1) and patient image (2) acquisitions are linked, and calibration/patient images should preferably be acquired using exactly the same protocol.

The reconstruction/correction step (3) leads from raw acquisition (counts) to activity in pixels/voxels composing the images. Even though the end product of step (3) may not lead *explicitly* to activity‐indexed images; it should be possible to derive activity in images at the end of this step (e.g., via a calibration factor).

The fourth step (4) allows the estimation of activity in regions or volumes of interest (VOIs), by segmenting and registering images acquired at different time‐points. Depending on the algorithms implemented, this fourth step leads to the quantification of activity present in the patient, in various organs/regions/tissues of interest or at the voxel level, at different time‐points.

Cumulated activities (also known as time‐integrated activities) (5) are defined as the integrals of the time–activity curves for the various VOIs. There are several ways to derive cumulated activity, depending on how the integration is performed between time‐points, how the extrapolation is performed and which model — if any — is used to fit time–activity curves (TACs) before integration.^33^, ^34^, ^35^, ^36^ Results obtained are the time‐integrated activity (TIA) (in Bq.s), or the time‐integrated activity coefficient (TIAC) (in s), obtained by dividing the cumulated activity by the activity administered to the patient *A_0_* (in Bq). Depending on the software, this can be done for VOIs or at the voxel level.

The absorbed dose calculation step (6) can be performed in different ways — using precomputed values (S values from specific absorbed fraction estimations)^37^ or by implementing different absorbed dose calculation algorithms (local energy deposition,^38^ convolution (using dose‐voxel kernels — DVK) in homogeneous or heterogeneous media,^39^, ^40^, ^41^ or Monte Carlo modeling of radiation transport^42^, ^43^, ^44^, ^45^, ^46^, ^47^). This can lead to different types of output, from average absorbed doses to absorbed dose maps and absorbed dose–volume histograms. Also of interest but not considered explicitly in Fig. 1 is the way dosimetric results are presented or used to provide extra information, such as absorbed dose rates or radiobiological indexes such as biological effective doses.

Splitting the clinical workflow into individual steps (as presented in Fig. 1) is relevant as currently available software applications do not address all the same parts of the dosimetric chain. Some allow raw data reconstruction, but stop after the calculation of the TIA(C) and therefore consider only steps 1–5. Others start after step 3, by considering activity maps (or equivalent, i.e., count‐indexed images + calibration factor). Some consider only the absorbed dose calculation part (step 6). In addition, some software only accepts a certain type of data (e.g., SPECT‐only). Therefore, a fair comparison of available software is a challenging task, since they address different parts of the workflow. It is therefore necessary to identify common criteria to perform this task. We present results of the comparison of five commercial software applications, based on the analysis of clinical datasets of patients who benefited from PRRT with ^177^Lu‐DOTATATE (LUTATHERA^®^), based on three evaluation criteria: organ masses, TIACs, and mean absorbed doses.

## 2 MATERIALS AND METHODS

### 2.A. Presentation of the dosimetry software platforms considered in the study

#### 2.A.1. **Dosimetry Toolkit** ^®^ **(DTK) from GE (Version 3.0423)**

DTK is an application in the Xeleris^TM^ software, which runs on Windows. The user can upload either serial anterior–posterior whole‐body scans (WBs); serial anterior–posterior WBs and one SPECT (or SPECT/CT) scan; or a minimum of three whole‐body serial SPECT (or SPECT/CT) scans. A calibration method is proposed, *based on planar measurements*, to estimate a calibration factor in cps·MBq^−1^. The dosimetry procedure is performed in two steps: first, the “preparation for dosimetry toolkit” is used for reconstruction of SPECT/CT raw data and definition of a reference for the registration (manual or automatic) of all CT scans; second, the “Dosimetry toolkit” application is used to segment (manually or automatically) different organs, create time–activity curves, and fit them using a mono‐exponential function, in order to calculate the TIAC for each organ. Finally, TIACs can be exported (by transcription) to OLINDA/EXM V1.0^48^ to compute absorbed doses.

#### 2.A.2. Hybrid dosimetry module™ (HDM) from HERMES (Version 1.0)

The HERMES software runs on Windows. It allows the reconstruction of imported raw data using HybridRecon‐Oncology version_1.3_Dicom (HROD) and contains the Hybrid Dosimetry Module™ (HDM). For example, HDM can accommodate a minimum of three serial anterior–posterior WBs, or three WBs and one SPECT (or SPECT/CT), or three serial SPECT (or SPECT/CT) scans. HDM requires a calibration factor in units of MBq·counts^−1^ (other options are available but not documented) obtained from SPECT/CT acquisitions. A calibration method is proposed, based on in‐house scans of a cylindrical phantom uniformly filled with radioactivity. Manual and automatic registration and segmentation can be performed. Fitting can be done using mono‐exponential or bi‐exponential functions. TIACs are computed at the organ/macroscopic tissue scale for selected ROIs/VOIs. Results are exported automatically to OLINDA/EXM V2.0^37^ which is integrated into HDM.

#### 2.A.3. STRATOS from Phillips (Imalytics 3.2, Rev 6289(64))

STRATOS is part of the IMALYTICS Research Workstation and runs on Windows. STRATOS uses reconstructed 3D SPECT/CT data. No calibration method is suggested by the company and the calibration factor is manually entered in units of Bq·Intensity^−1^ (where intensity is the number of counts in the image). Manual and automatic registration and segmentation can be performed. TIACs are calculated at the voxel level using the trapezoidal integration, and after the last time‐point, a mono‐exponential function assuming only physical decay is considered. Voxel‐based absorbed dose calculation is performed by convolution of dose voxel kernels (DVK), thereby generating absorbed dose–volume histograms (DVHs), TIACs, and mean absorbed doses.

#### 2.A.4. PLANET^®^ Onco Dose (PDOSE) from DOSIsoft (version 3.1.1)

Planet^®^ Onco Dose (PDOSE) runs on Linux. PDOSE was initially developed for the dosimetry of radioactive ^90^Y microspheres for the treatment of liver cancers.^49^ PDOSE only accepts reconstructed SPECT/CT (3D) datasets. No calibration method is suggested by the manufacturer; however, the calibration factor can be expressed in Bq·counts^−1^ or other options. It is also possible to define a calibration factor for every time‐point. Registration and segmentation can be performed (manual or automatic) and the software estimates mean TIA in VOI. Fitting can be done using a range of approaches — the trapezoidal method (with a variant that includes a physical decay mono‐exponential extrapolation for the tail of the curve), "X"‐exponential, mono‐exponential, bi‐ or tri‐exponential fits (currently eight fitting models are available). The mean absorbed doses can be calculated with or without media density correction, using either the local energy deposition^38^ or convolution of DVK.^39^, ^40^, ^50^ Fitting/integration of activity/absorbed dose rate can also be performed at the voxel level, generating DVHs (but this option was not validated for clinical applications at the time of the study).

#### 2.A.5. **SurePlan**™** MRT from MIM (Version 6.9.3)**

SurePlan™ MRT (from hereafter called “MRT”) from MIM is a software application that can be installed either in Windows or MacOS environments. An extra module of MRT allows the reconstruction of imported raw data under the same platform; however, we did not have access to that module for our study. MRT works using different workflows, allowing the user to work with 3D or hybrid datasets. The calibration method is similar to that of HERMES. The calibration factor can be expressed in different units depending on user needs in our case, MBq·counts^−1^. Manual and automatic registration (rigid or elastic) and segmentation can be performed using different tools. Fitting can be done using different approaches — the trapezoidal (including tail extrapolation), mono‐exponential or bi‐exponential fit — and there is an automatic option to choose the best‐fitting option per VOI.^51^ MRT also allows voxel‐based TAC fitting and integration. MRT estimates mean absorbed dose in VOI (also generating a DVH) by convolution of DVK. Fitting/integration results and DVHs can be obtained at the voxel level.

As a summary, for the software versions that were available at the time of this study, HDM and DTK supported only organ‐based dosimetry; STRATOS performed voxel‐based dosimetry (not validated for clinical applications); PDOSE and MRT could estimate the mean absorbed dose considering both organ‐based and voxel‐based approaches.

According to the steps outlined in Fig. 1, HDM and DTK address steps 2–6 (with an absorbed dose calculation step performed using precomputed S values using OLINDA V1.0 or V2.0 which can be included or not within the software platform), STRATOS and PDOSE consider steps 4–6, and MRT addresses steps 2–6 (but we could only test steps 4–6).

Even though some manufacturers recommend a procedure for the calibration process, we do not consider that any software deals with step 1, as none fully integrate the calibration process into the processing workflow.

### 2.B. Clinical data

Clinical data were obtained from patients treated with ^177^Lu‐DOTATATE at Institut Régional du Cancer de Montpellier (ICM).^52^ In that study, patients received four cycles of therapy, with a time interval between cycles of approximately 8 weeks. For each cycle, patients were administered approximately 7400 MBq. Activity measurements were performed with a radionuclide calibrator appropriately calibrated for ^177^Lu measurements. Images were acquired at ICM on a GE Discovery NM/CT 670 SPECT/CT, with a 9.5 mm crystal thickness and a medium energy general purpose (MEGP) collimator, and dosimetry was performed using DTK on the 12 patients for the first two cycles of the therapy.^52^

In our study, dosimetry was performed on a subset of two patients (one male, one female) for the first two cycles, considering only some selected organs; liver, spleen, and kidneys. Tumor and bone marrow dosimetry were not considered to bring any added value for the comparison exercise, as no specific methodology (e.g., partial volume effect correction) was implemented in any of the software studied. Patient characteristics are presented in Table I.

**Table I:** Characteristics of patients treated with ^177^Lu‐DOTATATE for PRRT.^52^

	Treatment cycle for female patient		Treatment cycle for male patient	
	First	Second	First	Second
Age (yr)	82		59	
Weight (kg)	57		79	
Height (cm)	153		180	
Injected activity (MBq)	7176.7	7239.4	7207.2	7188.2
Primary tumor (localization)	Pancreas NET		Small intestine NET	
Metastasis	Liver		Mesentery, liver	

### 2.C. Calibration procedure

For all software, a calibration factor is needed to convert the number of counts at the organ/VOI or voxel level to activity. For DTK, according to GE recommendations, the calibration factor should be obtained from planar acquisitions. It seemed strange to use a planar calibration factor for SPECT quantification, and therefore, we decided to implement as well a SPECT‐based calibration procedure for DTK. For HDM, the calibration factor must be obtained from SPECT acquisitions. For all other software, the calibration factor obtained from HDM was converted to the units required by each software platform.

#### 2.C.1. Planar acquisitions for DTK

A 16‐ml hollow sphere (external diameter 33.3 mm) filled with a homogeneous solution of ^177^Lu (75.8 MBq) was placed in air between the two heads. In order to assess the variability of the calibration factor, acquisitions were performed at different source–detector distances (8, 13, and 18 cm), taking the center of the sphere as a reference. Acquisition time was 300 s, with a matrix size of 128 × 128 and zoom = 1. For each distance, planar sensitivities were estimated from geometric mean (GM) images using ImageJ: (a) using a ROI covering the whole image, (b) drawing two circular ROIs, six and eight pixels in diameter (26.5 and 35.3 mm, respectively) centered on the maximal pixel count. No scatter correction was implemented for planar acquisitions. Planar sensitivity was extracted from Xeleris^TM^ and ImageJ, with variation with distance to the collimator, using the GM.

#### 2.C.2. SPECT/CT acquisitions

We used the NEMA phantom (body phantom NU2‐2001/2007) filled with water (no background activity). A fillable bottle (500 ml) simulating a kidney with 0.54 MBq·ml^−1^ of ^177^Lu solution was fixed inside the phantom. The acquisition time per projection was 120 s. One FOV could cover the whole phantom, with 60 projections in total, zoom 1, matrix size 128 × 128, two energy windows centered at 208 keV (±10%) and 177 keV (±5%), step & shoot, and body auto‐contour. CT acquisition protocol was as follows: 120 kV, automatic mA regulation, matrix size 512 × 512, noise index 6.4, slice thickness 5 mm, pitch 1.375, and standard reconstruction filter.

Additional phantom acquisitions performed at ICM were processed at Centre de Recherches en Cancérologie de Toulouse (CRCT). The same fillable sphere used for planar acquisitions was placed in a cylindrical phantom, empty or filled with water as proposed by Wevrett et al.^53^ The camera and acquisition protocol were the same, except for acquisition time (changed to 45 s per projection).

One set of images was reconstructed at ICM using the Xeleris^TM^ workstation and the same set of images was reconstructed at CRCT using HERMES‐HybridRecon‐Oncology version_1.3_Dicom (HROD). Both centers reconstructed raw data images as follows: OSEM (six iterations, ten subsets), all correction methods available in each workstation (scatter, attenuation, and collimator–detector response correction) and Gaussian post‐filter (set at 0.1 cm). Each center estimated the calibration factor by selecting a VOI of 500 ml (for the fillable bottle) using Nuclear Medicine (NM) images. At CRCT, the calibration factor for the fillable sphere was also calculated using NM images and the same reconstruction settings.

### 2.D. Patient acquisition protocol

Single photon emission computed tomography/computed tomography images centered on the abdominal region were acquired at 4, 24, 72, and 192 h post administration. In total, 60 projections (45 s per projection) acquired, with zoom 1, matrix size 128 × 128 (pixel size 4.42 mm), using two energy windows, one centered at 208 keV ± 10% and one at 177 keV ± 5%, step & shoot, and body auto‐contour. CT acquisition parameters for the first time‐point were: 120 kV, automatic mA regulation with a max = 200 mA, matrix size 51 × 512, noise index 6.43, time rotation of 0.8 s, pitch 1.375, slice thickness 5 mm, and standard reconstruction filter. For the other time‐points, rotation time of 0.6 s and 80 mA fixed was used.

### 2.E. Reconstructions

Reconstructions were performed using DTK and HROD modules.

#### 2.E.1. DTK

Transverse slices were reconstructed at ICM, as presented in Santoro et al.^52^ using “preparation for dosimetry Toolkit application,” including manufacturer's corrections such as DEW scatter using the 177 keV window, CT‐based attenuation, and collimator–detector response. Images were reconstructed using OSEM (six iterations, ten subsets) with a Gaussian post‐filter set at 0.1 cm. NM images generated by DTK had voxel sizes of 4.42 × 4.42 × 4.42 mm^3^.

#### 2.E.2. HERMES

The reconstruction was performed at CRCT using HROD with the main energy window only and applying corrections from the manufacturer — Monte Carlo‐based scatter correction,^54^ CT‐based attenuation correction,^55^ and collimator–detector response correction. Images were reconstructed using OSEM (six iterations, ten subsets) with a Gaussian post‐filter set at 0.1 cm. NM images generated by HROD had voxel sizes of 4.42 × 4.42 × 4.42 mm^3^. The SPECT standard uptake value (SUV) option^56^ was not available at the time of the study.

### 2.F. Data processing

A preliminary observation during our study was that the diversity of processing characteristics would eventually prevent any software comparison or benchmarking. In order to proceed, we decided to favor a common processing procedure whenever possible. Obviously, this choice may be felt as limiting for some software that proposes alternate possibilities. However, this is the only way to allow the comparison between software.

#### 2.F.1. DTK

Reconstructed images from “preparation for dosimetry Toolkit application” were uploaded in the “Dosimetry toolkit” application. Automatic rigid registration (translation and rotation) was performed taking the first SPECT/CT as reference. Liver, spleen, and kidneys were manually segmented using the first CT and NM images. Despite the fact that liver often contained several tumor metastases, as is frequent in this pathology,^15^ we decided to consider the liver volume of interest as a whole (as this should not limit the comparison of the results between software platforms). VOIs were then replicated over all time‐points, keeping the volume constant with time. The SPECT/CT calibration factor was used to derive the activity per organ at each time‐point. The administrated activity and date/time of administration were also entered at that stage. Fitting was performed using a mono‐exponential function, as available in DTK. By integrating fitted data, the software estimated the TIAC per organ. The goodness of the fit was assessed visually. Then, TIACs were transcribed to OLINDA/EXM V1.0. Mean absorbed dose calculation was performed using OLINDA/EXM V1.0 with patient organ mass adjustment.

#### 2.F.2. Hybrid dosimetry module™ (HDM)

Reconstructed images from the HROD module were uploaded into HDM. Automatic rigid registration was performed taking the first SPECT/CT as reference. Manual segmentation was carried out on each CT slice (ROI) to create a VOI for each organ. The software automatically replicates each VOI from the CT on the NM matrix. Segmented organs were copied onto other SPECT/CT series. If the generated contours did not match the position in axial plane of the SPECT/CT series, manual adjustment (by moving the ROI) was performed, but VOI volume was kept constant. The calibration factor in MBq·counts^−1^ along with the information regarding date/time and of the activity administration was entered at this stage. From t = 0 to the first data point, curve fitting based on trapezoidal method was done by assuming that activity was constant between time zero and the first acquisition time. From the first to the last time‐point, a bi‐exponential fit was done, using the Levenberg–Marquardt algorithm.^7^ From the last point to infinity, a mono‐exponential decay considering only physical half‐life was assumed (T_1/2p_ = 159.53 h).^57^ The goodness of the fit was assessed visually. Then, TIACs were computed and automatically exported to OLINDA/EXM V2.0. Mean absorbed doses were calculated using OLINDA/EXM V2.0 with patient organ mass adjustment.

#### 2.F.3. STRATOS

Reconstructed images from the HROD module were uploaded into STRATOS. Information regarding the calibration factor, in Bq·intensity^−1^, date/time of injection, and injected activity were entered. Automatic rigid registration was performed between the NM study of each day and the first CT study, taken as the reference. Segmentation was done in the same form as in HDM, but the software does not allow ROI replacement. STRATOS generated TIA maps with voxel sizes of 4.42 × 4.42 × 4.42 mm^3^, meaning that no resampling was necessary. Fitting was performed at the voxel level, assuming a straight line between zero and the first time point and a trapezoid integration between time‐points. After last time‐point, mono‐exponential integration (called “tail integration”) was performed, considering only physical half‐life. TIA was then calculated for each voxel. Three‐dimensional TIA maps were convolved with a precalculated water DVK with the same spatial sampling to obtain 3D absorbed dose maps under the assumption of homogeneous propagating medium^58^ of water density.

#### 2.F.4. PDOSE

Reconstructed images from the HROD module were uploaded into PDOSE. Segmentation was carried out manually as for HDM and STRATOS. The software automatically created the VOI from the CT on the NM matrix. Note that in this case, segmentation was performed before registration.

Using the segmented structures, automatic rigid registration was performed and optimized organ by organ, taking the first SPECT/CT series as a reference. Registered images were saved in a new space, called “registered space.” When the registration process was done, rigid propagation of the structures occurred in the registered space. In our study, the generated volumes were therefore kept constant among all images in the registered space. The calibration factor was included at this stage in units of Bq·counts^−1^, along with the injection date/time information. Fitting in PDOSE can be done using different options (mono‐, bi‐, tri‐exponential, trapezoidal, etc.), and the evaluation of the goodness of the fit can be performed visually and by using the Spearman coefficient. We used both criteria to choose between fitting options. The integration of fitted data yielded TIA and TIAC (TIA was divided by injected activity) calculated for the organs considered in the study. The mean absorbed dose calculation was based on local energy deposition, with density correction.

#### 2.F.5. MRT

Reconstructed images from the HROD module were uploaded into MRT. VOIs generated with PDOSE were exported as DICOM RT‐Struct files and then imported into MRT, thanks to the DICOM RT‐Struct supports available in both software, in order to have exactly the same VOIs for the two software applications. Segmentation was not performed because all structures were imported using DICOM RT‐Struct. Automatic registration was performed. In our study, the generated volumes were kept constant among all time‐points. Using the segmented structures, automatic rigid registration using the whole field of view (FOV) was performed, taking the first CT series as a reference. The software does not allow repositioning of each structure, but the whole FOV can be moved in order to match one particular structure. The calibration factor was in units of Bq·counts^−1^ (this was an option defined in agreement with MIM’s representative). The injection date/time information and administrated activity were entered. TACs were generated using an automatic tool.^51^ TIACs were calculated for the organs considered in the study and saved.

Absorbed dose calculation was performed using the 3D cumulated activity maps. These maps were convolved with a precalculated DVK (with density correction) at the same spatial sampling, thereby providing a 3D absorbed dose map. The mean absorbed dose for each VOI was reported based on the absorbed dose map.

For all software, organ masses were estimated using density values from the GATE code,^59^ widely used for Monte Carlo dosimetry in our team, assuming 1.06 g·cm^−3^ for liver and spleen and 1.05 g·cm^−3^ for the kidneys.

## 3 RESULTS

### 3.A. Calibration

#### 3.A.1. Planar

**Fig. 2:**
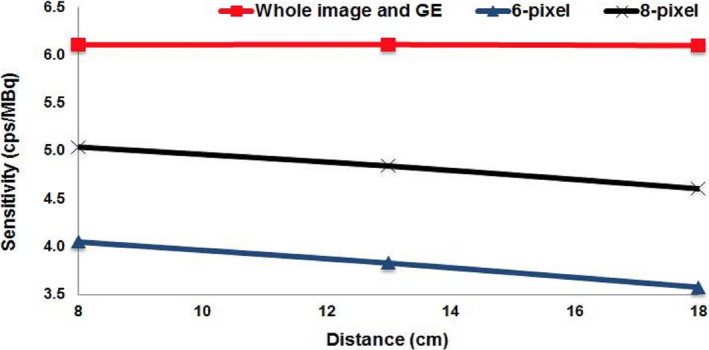
Planar sensitivity variation with source to collimator distance for GE‐Discovery NM/computed tomography 670. Red line was generated using the whole image. Blue and black lines were generated using two circular ROIs different in diameter. [Color figure can be viewed at wileyonlinelibrary.com]

Sensitivity results for GM can be seen in Fig. 2. Two ROI sizes were considered (six and eight pixels). The plots in this figure show sensitivity changes with source to collimator distance (SCD) analyzed with ImageJ. For the eight pixels‐ROI, the sensitivity varied from 5.0 to 4.6 cps·MBq^−1^. For the six pixels‐ROI, the sensitivity varied from 4.0 to 3.6 cps·MBq^−1^. Using Xeleris^TM^ to process the same images and placing a ROI covering the whole image, the same sensitivity value (6.1 cps·MBq^−1^) was obtained for all SCD.^52^

#### 3.A.2. SPECT

For the NEMA phantom geometry (bottle), the SPECT calibration factor was 5.67 cps·MBq^−1^ when calculated using DTK reconstruction (i.e., close to the values obtained via planar calibration), whereas that obtained from HDM was 13.6·10^−6^ MBq·counts^−1^, equivalent to 10.21 cps·MBq^−1^. For the sphere source, using HDM reconstruction, the average calibration factor was 38.3·10^−6^ MBq·counts^−1^ (approx. 9.68 cps·MBq^−1^).

### 3.B. Clinical data results

**Fig. 3:**
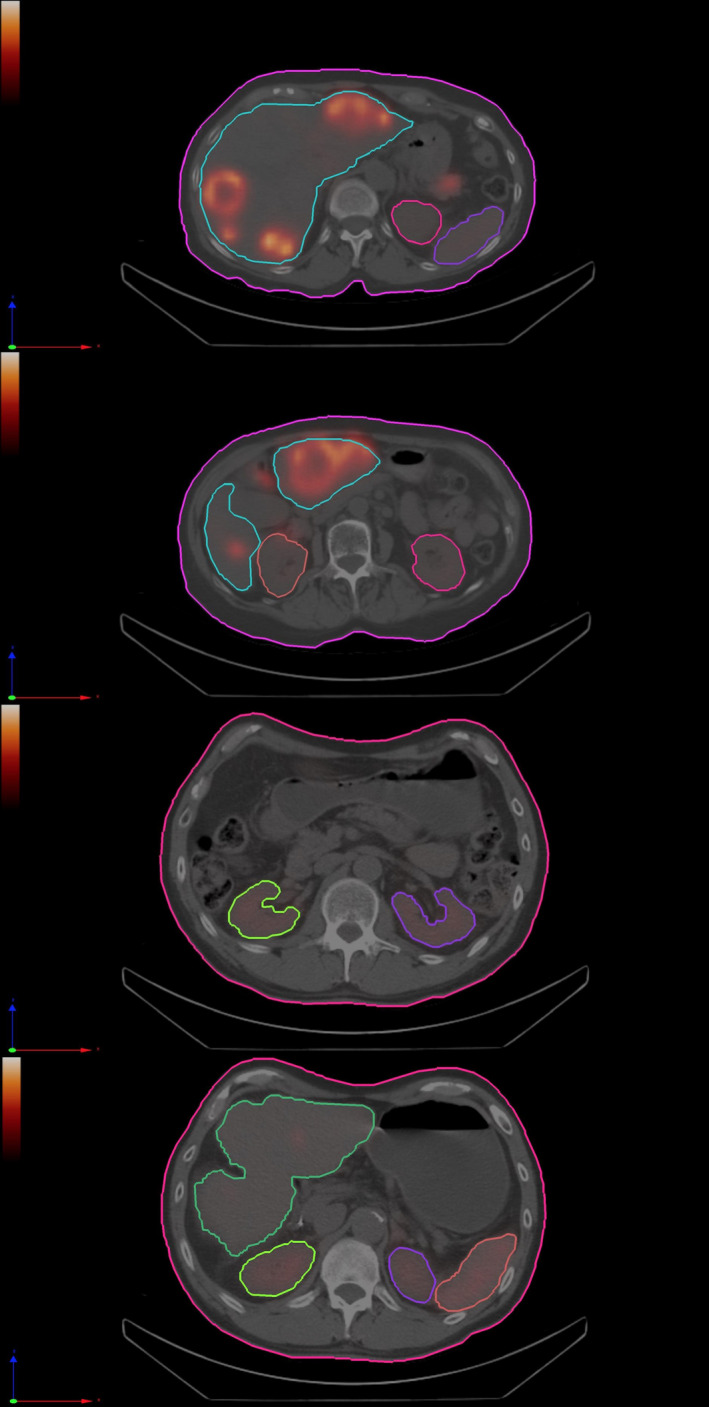
Two different axial slices showing segmented organs (liver, spleen, and kidneys) in the reference computed tomography, using PDOSE workstation (image reconstruction was performed using HROD). (a), (b) female patient first treatment cycle; (c), (d) male patient first treatment cycle. [Color figure can be viewed at wileyonlinelibrary.com]

Segmentation of studied organs for male and female patients using PDOSE can be seen in Fig. 3. Images are from the reference SPECT/CT. In the case of the female patient, several metastases can be visualized in the liver. For the male patient, liquid is observed within the stomach.

Table II shows the average masses obtained for liver, spleen, and kidneys (in grams) estimated by segmentation for each software platform, for both patients, and the two treatment cycles.

**Table II:** Mean and standard deviation of organ masses among all five dosimetry software platforms.

Organ	Patient	Cycle	Mean (g)	Range (g)	Std Dev (g)
Liver	Female	Cycle 1	1627.7	1560.3–1656.5	38.32
		Cycle 2	1550.4	1476.6–1643.0	59.62
	Male	Cycle 1	1344.3	1208.4–1397.6	77.10
		Cycle 2	1300.8	1282.6–1306.3	10.22
Spleen	Female	Cycle 1	104.5	99.0–112.4	6.04
		Cycle 2	104.9	98.2–116.6	7.74
	Male	Cycle 1	241.4	234.0–254.4	8.55
		Cycle 2	248.2	234.5–272.4	17.06
Kidneys	Female	Cycle 1	273.1	270.8–279.3	3.57
		Cycle 2	273.6	266.6–294.0	13.72
	Male	Cycle 1	431.3	390.7–482.0	40.09
		Cycle 2	435.9	402.7–483.0	38.94

**Fig. 4:**
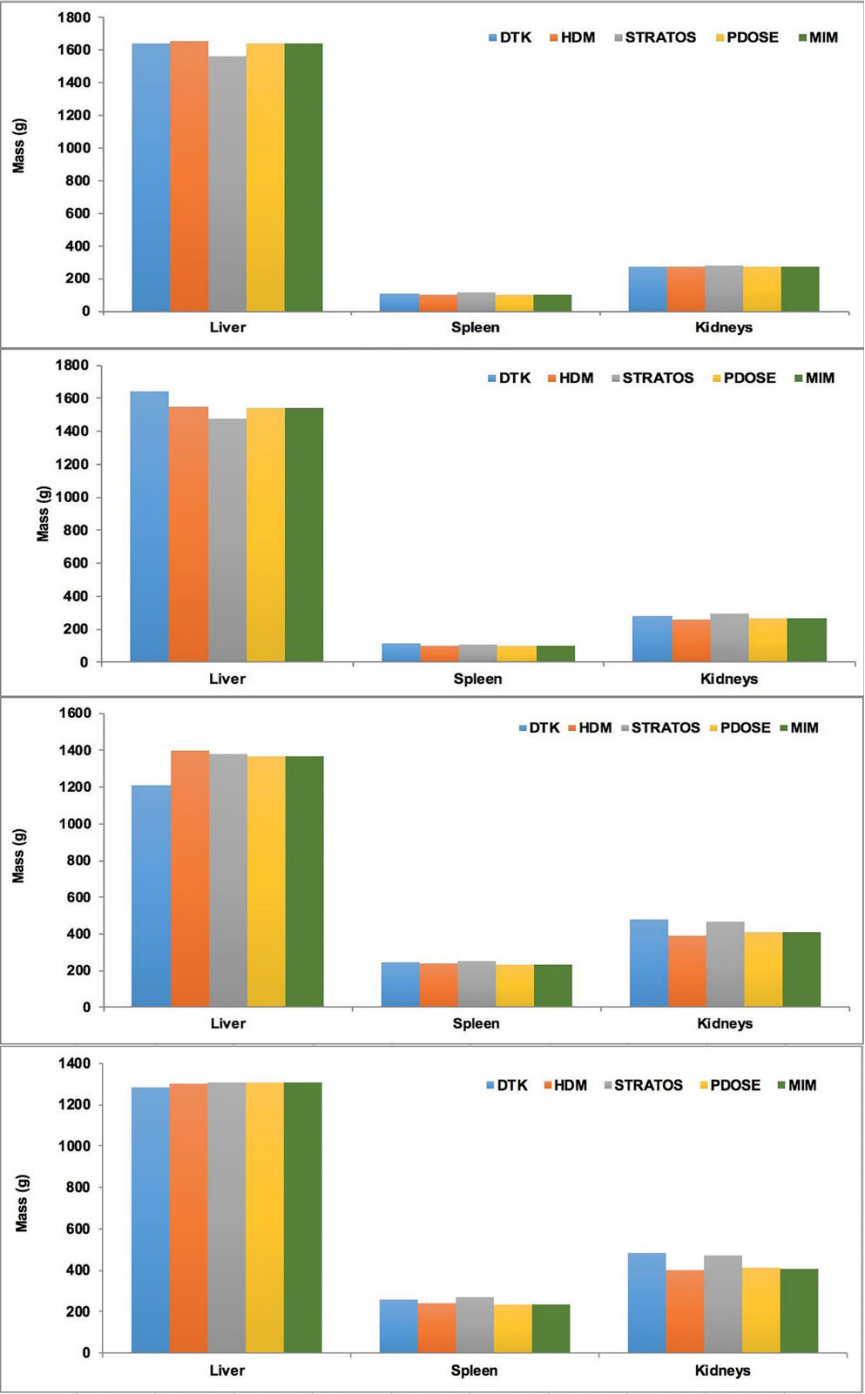
Organ masses after manual segmentation for liver, spleen, and kidneys. Female patient: (a) first treatment, (b) second treatment. Male patient: (c) first treatment, (d) second treatment. [Color figure can be viewed at wileyonlinelibrary.com]

In Fig. 4, masses for organs segmented using each software platform can be seen, showing that the segmentation processes generate volumes that have similar values. The most significant variability can be seen in the liver, but the relative differences between masses obtained using different software platforms are always <9.5%.

TIACs were evaluated using the following calibration factors: 5.67 cps·MBq^−1^ for DTK, 38.3 × 10^−6^ MBq·counts^−1^ for HDM, 38.3 Bq·intensity^−1^ for STRATOS, and 38.3 Bq·counts^−1^ for PDOSE and MRT (note the difference in units for each workstation). The calibration factors for HDM, STRATOS, PDOSE, and MRT were corrected taking into account the fact that patient image acquisitions were performed with 60 projections in total and 45 s per projection, in comparison to the phantom bottle acquisition (60 projections in total and 120 s per projection). The sensitivity values (images reconstructed using HROD) in cps·MBq^−1^ used for HDM, STRATOS, PDOSE, and MRT are of the same order of magnitude as those reported by other authors^60^, ^61^, ^62^ for ^177^Lu.

**Fig. 5:**
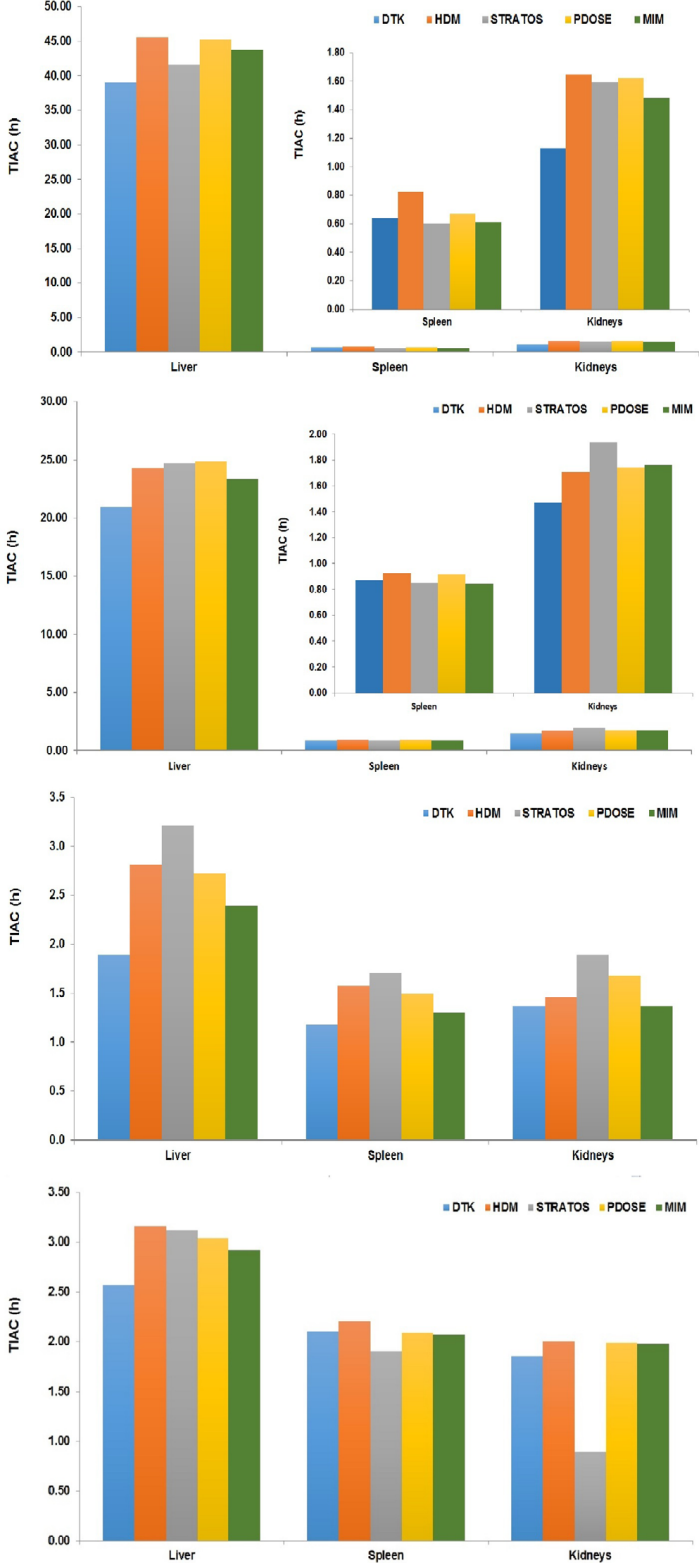
Time‐integrated activity coefficient for liver, spleen, and kidneys. Female patient (for spleen and kidneys, a zoom‐in is shown in the upper right part): (a) first treatment, (b) second treatment. Male patient: (c) first treatment, (d) second treatment. [Color figure can be viewed at wileyonlinelibrary.com]

TIACs obtained with the different software platforms are presented in Fig. 5. In this case, segmentation, registration, and fitting steps have a direct impact on the results. For the female patient, for both cycles, results are very close regardless of the software platform used.

The major difference can be seen for kidneys, first cycle. In the case of the male patient, a similar tendency is observed, but large differences can be seen in the case of the liver (first treatment cycle) and the kidneys (second treatment cycle).

Table III presents the average TIAC for the liver, spleen, and kidneys with associated standard deviation. On average, the relative standard deviation between TIAC obtained from different software platforms is <16% for each cycle, and equal to 12%, for all cycles considered. However, in some situations, the relative standard deviation can be quite high (i.e., male patient, cycle 2 kidneys: 28.2%), a variation largely induced by large discrepancies in the TIACs obtained by STRATOS vs the other software [Fig. 5(d)].

**Table III:** Mean and standard deviation of TIACs among all five dosimetry software platforms.

Organ	Patient	Cycle	Mean (h)	Range (h)	Std Dev (h)
Liver	Female	Cycle 1	43.1	39.0–45.7	2.75
		Cycle 2	23.6	21.0–24.8	1.60
	Male	Cycle 1	2.6	1.9–3.2	0.49
		Cycle 2	3.0	2.6–3.2	0.24
Spleen	Female	Cycle 1	0.7	0.6–0.8	0.09
		Cycle 2	0.9	0.8–0.9	0.04
	Male	Cycle 1	1.5	1.2–1.7	0.22
		Cycle 2	2.1	1.9–2.2	0.11
Kidneys	Female	Cycle 1	1.5	1.1–1.7	0.21
		Cycle 2	1.7	1.5–1.9	0.17
	Male	Cycle 1	1.6	1.4–1.9	0.23
		Cycle 2	1.7	0.9–2.0	0.48

**Fig. 6:**
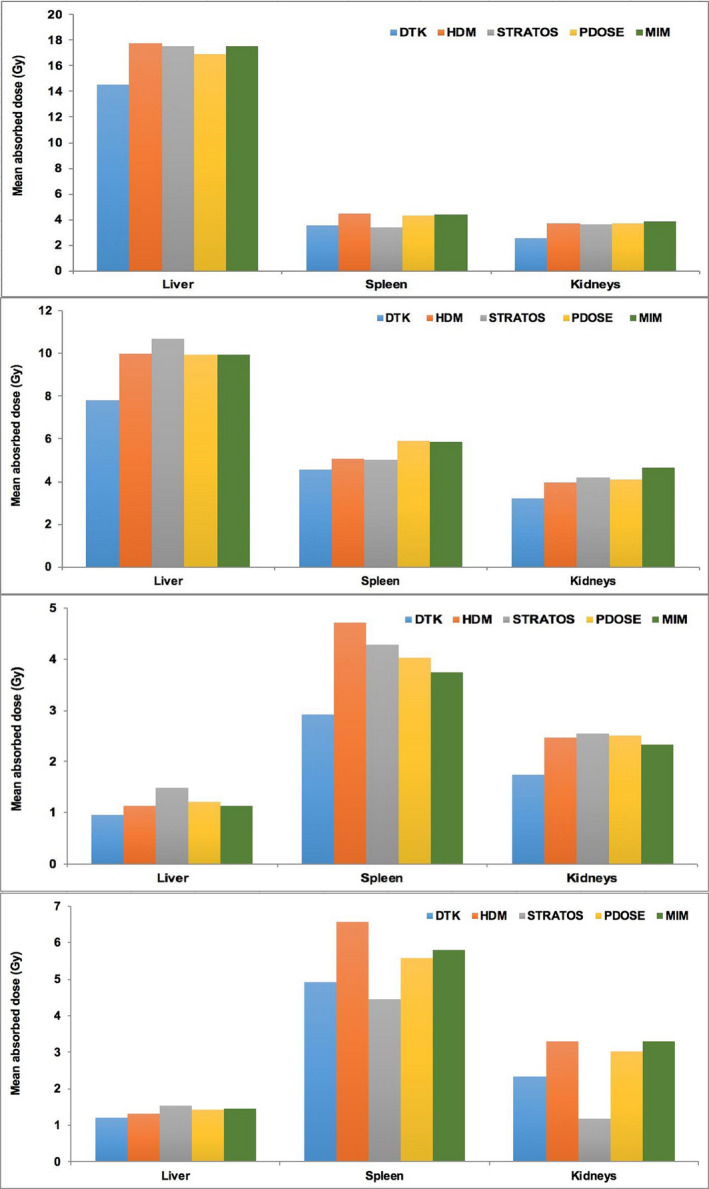
Mean absorbed dose (corrected by organ mass) results among all software for liver, spleen and kidneys. Female patient: (a) first treatment, (b) second treatment. Male patient: (c) first treatment, (d) second treatment. [Color figure can be viewed at wileyonlinelibrary.com]

Mean absorbed doses obtained with the different software platforms are presented in Fig. 6. In the case of the female patient, for each organ, the results are close to each other. Similar behavior can be seen for the male patient except for spleen (both treatment cycles) and kidneys for second treatment cycle. Comparing DTK and HDM results, HDM produces slightly higher results than DTK (but the two versions of OLINDA provide different S‐values).

It can be seen that the results are in general quite close across software platforms (except for the kidneys cycle 2, male patient), and at least of the same order of magnitude, despite the range of absorbed dose calculation solutions/algorithms. In general, the observed differences in absorbed doses followed those observed in TIAC (Fig. 5) for the majority of the organs/software platforms.

Table IV presents the mean absorbed doses obtained for the liver, spleen, and kidneys and associated standard deviation. Relative standard deviations in mean absorbed doses, on average are <16%, with a maximum at 41% (for the kidneys cycle 2, male patient) and increasing slightly when compared to those observed for TIAC. The mean absorbed doses to the kidneys are of the same order of magnitude as those reported by other authors.^1^, ^63^, ^64^, ^65^, ^66^, ^67^

**Table IV:** Mean and standard deviation of absorbed dose among all five dosimetry software platforms.

Organ	Patient	Cycle	Mean (Gy)	Range (Gy)	Std Dev (Gy)
Liver	Female	Cycle 1	16.8	14.5–17.8	1.35
		Cycle 2	9.5	7.8–10.7	1.25
	Male	Cycle 1	1.2	1.0–1.5	0.19
		Cycle 2	1.4	1.2–1.5	0.13
Spleen	Female	Cycle 1	4.0	3.4–4.5	0.50
		Cycle 2	4.9	4.6–5.9	0.56
	Male	Cycle 1	3.9	2.9–4.7	0.67
		Cycle 2	5.3	4.4–6.6	0.92
Kidneys	Female	Cycle 1	3.5	2.5–3.8	0.54
		Cycle 2	3.8	3.2–4.7	0.45
	Male	Cycle 1	2.3	1.7–2.6	0.34
		Cycle 2	2.3	1.2–3.3	0.95

## 4 DISCUSSION

During this study, five commercially available dosimetry software platforms were evaluated. In two of them, it was possible to perform SPECT reconstruction (we did not have access to that option in MIM). Therefore, issues associated with the impact of reconstruction on quantification for every solution were not addressed.

### 4.A. Planar calibration

Our sensitivity results, despite some differences in experimental settings, are of the same order of magnitude as those presented by other authors.^61^, ^62^ Yet, as the dosimetric studies presented here are based on SPECT/CT images, we decided (contrary to GE DTK recommendations) to use a SPECT‐based calibration factor. The comparison of planar vs SPECT‐based calibration factors is not strikingly different, and our choice seems more consistent with a global (calibration + analysis) dosimetric workflow.

### 4.B. SPECT calibration

The SPECT calibration factors obtained from DTK and HROD were different, even though they were obtained from the same raw data (projections). The important difference relates to the reconstruction algorithm implemented within the two platforms (Xeleris and HERMES) and the correction methods applied. The standard uptake value option (SUV) in HERMES was not included with our license, and according to HERMES, this is the option required to compare their reconstruction results with other manufacturers. Even though OSEM is used in both workstations, its implementation and associated corrections differ.

Yet, it must be noted that even though the reconstruction process impacted calibration factors (3.A.2), this had little impact on activity determination (as long as calibration *and* patient projections were reconstructed consistently by the *same* software, quantification yielded the same results regardless of the platform). This clearly indicates why it is important to acquire, reconstruct, and process data in the same way and using the same software for calibration and clinical studies.

Therefore, as recommended in MIRD 23,^68^ SPECT calibration should be performed to characterize the sensitivity of the system, using large sources to avoid partial volume effect (PVE). Planar acquisition may be used for QC purposes, before patient acquisition, just to verify the stability in sensitivity of the SPECT system.

To conclude on the calibration, it is striking to see that the software platforms considered in our study expect calibration factors to be expressed so differently. In addition, some expect the same calibration factor for all time‐points, thereby forbidding changes in acquisition duration. As this is a major source of potential mistakes, we consider that the calibration phase (step 1 in Fig. 1) and patient data acquisition (step 2) should be explicitly linked, for example, in a “calibration specific module.” This is certainly an important point to address in future versions of commercial dosimetric software.

### 4.C. Clinical data

Several articles already reported on patient dosimetry of ^177^Lu‐DOTATATE.^1^, ^5^, ^8^, ^11^, ^63^, ^64^, ^65^, ^66^, ^67^, ^69^, ^70^, ^71^, ^72^ Our objective was to assess the performance of existing commercial solutions for dosimetry purposes, which is why we selected a reduced number of clinical cases and organs, and did not considered tumors or image‐based bone marrow dosimetry.

In addition, our comparison was performed using software (and versions) available at the time, and our results may have to be revised in time: commercial dosimetry software platforms is still in its infancy and manufacturers tend to update the software version quite frequently.

Only kidneys, spleen, and liver were chosen in order to avoid having to implement PVE corrections. Indeed, the clinical dataset used is a subset of a dosimetry study that considered PVE corrections (using recovery coefficients) for small structures.^52^ Still, we feel that PVE correction would not dramatically change the results of our comparison. This is because its implementation would be the same for all dosimetric procedures/solutions considered in the study — as none of the studied software currently implements PVE corrections in the context of clinical dosimetry. This is also a domain that calls for improvements, especially when tumor dosimetry is implemented (i.e., a range of volumes and shapes that may suffer — or not — from PVE).

As a conclusion, a continuation of this project should consider a more extended study. More patient datasets and more radiopharmaceuticals should be considered. In addition, since future versions of the dosimetry software platforms may implement improved methodological developments, various aspects of clinical dosimetry (hybrid approaches), and broader target range (tumor, bone marrow) should also be considered.

One of the main challenges in our comparison was to identify common checkpoints among all software platforms since the proposed workflows are so different between manufacturers. The organ masses, TIACs, and absorbed doses were provided by all five platforms. Ideally each step of the dosimetric workflow should have a method to assess its performance. So far, none of the solutions evaluated proposed a full evaluation of the uncertainties associated with each step. This is a development which needs to be addressed in the future.

#### 4.C.1. Organ mass

The masses of the organs (kidneys, spleen, and liver) obtained by the various software considered in this study were very similar. Organ masses resulting from DTK were the reference data. We only considered relatively large organs, and also for three software platforms (HDM, STRATOS, and PDOSE), the same operator performed all steps to avoid user variability in volume/mass determination. In the case of MRT, organ masses obtained were identical to those obtained with PDOSE, due to the use of the same VOIs shared as RT‐Struct files. All systems tested provided close results for large organs; however, the determination of smaller tumor masses is more challenging — but probably at this stage equally challenging for all software.

#### 4.C.2. Registration

The registration process is normally carried out using CT reconstructed images within the whole nuclear medicine FOV. A registration process performed organ by organ is promising in terms of reproducibility.^73^ Most software platforms propose a rigid registration, but in some cases, elastic/deformable registrations are also available. The rigid registration is mainly performed taking bone structures within the patient as a reference, but this does not consider the variation in the patient positioning between consecutive SPECT/CT scans, movement of the patient between CT and SPECT acquisitions, respiratory motion, patient tilt, and organs’ movements (due to respiratory motion or different placement). For example, in Fig. 3, it can be seen that liquid in the stomach changed the position of the spleen and left kidney. This may not be seen on images acquired at different times, when the stomach is empty. These aspects are all sources of error, which negatively affect the registration process. Therefore, the accuracy of image registration should be verified.^74^

Nuclear medicine would certainly benefit from features currently present in external beam radiotherapy departments. Unfortunately, the software platform included in our study just allows the user to visually validate the registration stage. No tool to evaluate or quantitatively assess the goodness of the registration is offered.

#### 4.C.3. Segmentation

In our study, slice‐by‐slice manual segmentation was performed for all software platforms. Manual segmentation is a user‐dependent process^75^ that should be validated. The segmentation of several structures was performed using the first SPECT/CT as a reference, and then, segmented structures were copied from the reference SPECT/CT to other time‐point SPECT/CT (propagation of the VOI).

The structure propagation process has a strong dependence on the registration stage. If there are errors in the registration procedure, the placement of the structure will change or may not fit from one time‐point SPECT/CT to another. HDM and PDOSE allow moving or modifying the size of the propagated structure. We decided to allow structure displacement (and/or registration optimization at the organ level) but to keep the size of each propagated structure constant. The idea was to keep organ volumes constant along the timeline, in order to calculate TIAC and to decrease the impact of operator‐dependent procedures.

In this study, we did not address the segmentation process itself and used only the segmentation tools provided with each software platform. It is clear that external solutions exist — for example, from external beam radiotherapy treatment planning systems — that allow a swift and efficient segmentation of the volumes of interest. Yet, their use in our domain is limited to the ability to import patient segmented datasets in DICOM RT‐Struct format, an important feature currently provided by DOSIsoft and MIM, while HERMES may propose that feature as an option.

#### 4.C.4. Time–activity curve fitting and TIAC determination

Each software platform provides different fitting models, including trapezoidal integration and/or mono‐ or bi‐exponential fit of time–activity curves. A large heterogeneity in proposed solutions was observed regarding the extrapolation before the first time‐point, the interpolation between time‐points, and the extrapolation after the last time‐point.

In STRATOS, the fitting is carried out by segments, using the trapezoid method between time‐points, and extrapolated by a single exponential with physical decay after the last time‐point. In the case of DTK, only mono‐exponential fitting can be done. In the case of HDM, we applied bi‐exponential fitting in almost all cases (mono‐exponential fitting was only performed for kidneys, in the first treatment cycle of the male patient). In the case of PDOSE, visual assessment as well as the Spearman coefficient were used to evaluate the goodness of the fit. The majority of times a mono‐exponential or bi‐exponential fit was used. Sometimes a function expressed as follows was used:

$$f\left(t\right)=At^{b}e^{‐ct^{d}}$$

where A, b, c, and d are fitting parameters.

Apart from the four SPECT/CT time‐point measurements, we also considered no activity at t = 0 h. In the case of MRT, an automatic tool^51^ was developed for that purpose.

Most software platforms computed organ‐based cumulated activity. The determination of voxel‐based (cumulated) activity is proposed by default by STRATOS; however, voxel‐based activity determination is challenging — and no tool is provided to validate the goodness of the fit in that context. This may explain why STRATOS results seem to be quite different from all the others in some cases [Figs. 5(c) and 5(d)].

At this stage, it is difficult to compare the performances of each software platform regarding time–activity curve fitting. The limited fitting options provided by some platforms prevented us from implementing the same approach for all systems. Further investigations based on identical activity maps are planned. This means that specific test datasets have to be developed. In addition, we intend to use NUKFIT^34^ to improve the definition of the most suitable fitting functions.

#### 4.C.5. Absorbed dose results

Having listed the many reasons why this should not be the case, the absorbed doses computed by the five different solutions were close for the majority of the organs, at least for the two cycles of two patients (four dosimetric studies) considered here. We previously presented a comparison of dosimetric results obtained using the same TIAC but different absorbed dose calculation procedures that demonstrated that this last step does not have a major impact on the results — at least for mean absorbed doses at the organ/macroscopic tissue level.^76^ A clear continuation of this study will consider voxel‐based absorbed dose calculations, a feature present or under development in most platforms but not validated for clinical applications yet. Absorbed dose–volume histograms will be compared rather than mean absorbed doses at the organ level, as this may probably allow a better discrimination between different radiation transport algorithms.

## 5 CONCLUSIONS

The objective of this study was to compare the results generated by five commercial dosimetry software platforms. Throughout the study, this proved to be quite difficult since all platforms consider different segments of the dosimetric workflow. However, the results obtained in terms of organ masses, TIAC, or absorbed doses were generally consistent between workstations, which is an encouraging result.

The objectives of this work are not to provide a ranking or to recommend a given solution, firstly because we cannot claim to have studied all available commercial solutions, but also as this is a recent and rapidly evolving field, where new software updates are regularly presented, with new features to further enhance their capabilities.

Still, at the end of this work, we can recommend features (a wish list) that appear to be desirable for a dosimetry software platform:

‐ Specific workflows should be available in order to accommodate a large spectrum of clinical applications that require different implementations of clinical dosimetry. This would improve the user‐friendliness of dosimetry software platforms (not specifically reported in our work, but for which improvements would be welcome for all solutions considered in this study!).
‐ Import/export features (in DICOM format) would be desirable to allow processing data from various systems. This would be helpful in the situation of a central processing of data acquired in different places. This should include the possibility to import/export segmented structures and absorbed dose maps (DICOM RT‐Struct and RT‐Dose).
‐ Despite the claims that commercial solutions allow performing dosimetric studies within significantly decreased times when compared with academic solutions, a remarkable time is still spent in the verification that acquired datasets conform to the expectations. Internal “sanity” checks should be performed automatically for each study, to make sure that images/data are acquired using the relevant protocol (spectrometry parameters, collimator, isotope, and to a lesser extent, acquisition duration).
‐ The calibration process should be well described, or even better, a “calibration module” should be available, where the calibration factor determined for the study would be passed from one step to another.
‐ A modular approach would be good, in order to allow step‐by‐step processing (providing checkpoints) or the possibility to perform a dosimetry study in different sessions (some solutions currently require performing a dosimetry study from the beginning to the end, as no saving of intermediary state is possible).
‐ In a related domain, the storing of intermediary results (segmentation, registration to name a few) and a history of the processes performed should be available to allow traceability and a retrospective processing of dosimetric studies.
‐ The output format should be standardized and at least well documented.
‐ Ideally, uncertainty analysis should be implemented within the workflow. Gear et al. recently presented the EANM practical guidance on uncertainty analysis for molecular radiotherapy absorbed dose calculations^77^ that considers the whole dosimetric chain. This gives the framework for future research.

This list highlights several domains of potential optimization — and in mirror reflects some of the limitations of currently available commercial software. However, the growing availability of user‐friendly clinical dosimetry solutions is promising in order to further develop and optimize targeted radionuclide therapy.

## 6 CONFLICT OF INTEREST AND FINANCIAL DISCLOSURES

The authors declare that they have not competing interest. Regarding the owned status of each software, **DTK** was purchased by ICM, **HERMES** and **STRATOS** were purchased by CRCT, **PDOSE** and **MRT** were lent free of charge by DOSIsoft and MIM, respectively, to CRCT for evaluation.

Manuel Bardiès is consultant for IPSEN; in the past, he acted as consultant for Roche and he also received honorarium from BTG and Bayer. Emmanuel Deshayes was invited in 2018 to attend an international meeting by AAA who sponsored registration fees and travel expenses. The rest of the authors have no conflicts to disclose.
